# Unemployed Women

**Published:** 1920-02-14

**Authors:** 


THE PUBLIC WELL-BEING—(*continued*).

### Unemployed Women.

The Women's Industrial League formed about a year ago has collected a considerable amount of useful information on problems of labour as they affect women. The League says the official figures give the number of unemployed women at 52,000, but it is practically certain that the true figure is nearly double this one, for the employment exchange returns do not cover all working women, besides leaving out of account altogether a large number of the professional and clerical women who are now looking for work. It seems deplorable that at a time when the country is in need of greater production and industrial activity, to compete successfully with other countries, there should be lying idle a vast amount of economic wealth—for the knowledge and skill acquired by women during the war, both by training and their own efforts, in the factory and the office, as well as in administrative and organising work, is surely wealth of a most valuable kind. Again, there are many women who had not earned their living before the war, who must now keep themselves, and sometimes dependants as well. There are the women who have become widows during the war, there are the widows whose private incomes are insufficient to-day, owing to the increased cost of living; there are the young girls who did not expect to have to work, and are now compelled by circumstances to earn their living, and there are the girls who lost the training they would have had by going straight from school into various kinds of war work, and are now unfitted for peace-time occupations.

As regards wages, the League notes that the ratio of women's rates to men's rates in any one trade tended generally to increase during the war, but that after it the women's ratio began to fall and gives indications of going lower still.

